# Self-healing polysaccharide-based hydrogels as injectable carriers for neural stem cells

**DOI:** 10.1038/srep37841

**Published:** 2016-11-29

**Authors:** Zhao Wei, Jingyi Zhao, Yong Mei Chen, Pengbo Zhang, Qiqing Zhang

**Affiliations:** 1 State Key Laboratory for Strength and Vibration of Mechanical Structures, International Center for Applied Mechanics and School of Aerospace, Collaborative Innovation Center of Suzhou Nano Science and Technology, Xi’an Jiaotong University, Xi’an 710049, China; 2 Department of Anesthesiology, The Second Affiliated Hospital of Xi’an Jiaotong University, Xi’an 710003, China; 3 Institute of Biomedical and Pharmaceutical Technology, Fuzhou University, Fuzhou 350002, China, Fujian Guided Tissue Regeneration (GTR) Biotechnology Co., Ltd., Fuzhou 350108, China

## Abstract

Self-healing injectable hydrogels can be formulated as three-dimensional carriers for the treatment of neurological diseases with desirable advantages, such as avoiding the potential risks of cell loss during injection, protecting cells from the shearing force of injection. However, the demands for biocompatible self-healing injectable hydrogels to meet above requirements and to promote the differentiation of neural stem cells (NSCs) into neurons remain a challenge. Herein, we developed a biocompatible self-healing polysaccharide-based hydrogel system as a novel injectable carrier for the delivery of NSCs. N-carboxyethyl chitosan (CEC) and oxidized sodium alginate (OSA) are the main backbones of the hydrogel networks, denoted as CEC-l-OSA hydrogel (“l” means “linked-by”). Owing to the dynamic imine cross-links formed by a Schiff reaction between amino groups on CEC and aldehyde groups on OSA, the hydrogel possesses the ability to self-heal into a integrity after being injected from needles under physiological conditions. The CEC-l-OSA hydrogel in which the stiffness mimicking nature brain tissues (100~1000 Pa) can be finely tuned to support the proliferation and neuronal differentiation of NSCs. The multi-functional, injectable, and self-healing CEC-l-OSA hydrogels hold great promises for NSC transplantation and further treatment of neurological diseases.

Transplantation of neural stem cells (NSCs) is a promising therapeutic strategy for the treatment of neurological diseases^1^,^2^,^3^. Transplanted NSCs are expected to proliferate and differentiate in the lesion cavity, and eventually integrate with the host tissue to promote neural regeneration. Biomaterial-based cell delivery systems which loaded NSCs within biocompatible and biodegradable soft materials, are explored as an important strategy for increasing cell population, promoting differentiation and prolonging cell function *in vivo*^4^,^5^,^6^,^7^.

Soft and biocompatible hydrogel, possessing three-dimensional (3D) polymer networks and physicochemical properties similar to the extracellular matrices of tissues^8^,^9^,^10^,^11^, can provide the 3D microenvironment for the differentiation and engraftment of transplanted NSCs. Injectable hydrogel, delivering cells into localized lesion site within any defect shape via minimally invasive manner, has been extensively applied in the delivery of NSCs^12^,^13^,^14^. In the case of conventional injectable hydrogels used for the transplantation of NSCs, both the cell suspension of floating cells and hydrogel precursors are injected into the target site simultaneously, then the cells are embedded into the polymer networks of the hydrogels after gelation. Unfortunately, the conventional injectable hydrogels without self-healing ability may be easily damaged by external forces which further seriously deteriorate their functionalities. Therefore, self-healing hydrogels, which can automatically restore their integrity of network structures and functionalities after damages without the need of external interventions, have been explored to meet the needs of biomedical applications^15^,^16^,^17^,^18^. Currently, the self-healing injectable hydrogels, possessing the dual capabilities of self-healing and injection, are emerging as a new generation of multifunctional vehicles for cell therapy^19^,^20^,^21^. Different from the conventional injectable hydrogels, self-healing injectable hydrogels can directly deliver cells via transplanting the cell-loaded hydrogel fragments which can self-heal into integral connected structures after filling a lesion site.

Self-healing and injectable hydrogels can provide several advantages over the conventional injectable hydrogels in cell delivery^22^,^23^, including: (i) avoiding the potential risks of cell loss during the injection, as well as providing necessary mechanical protections for the delivered cells from the shear damage during the injection^24^,^25^; (ii) confirming the morphologies and functionalities of the cells loaded inside the 3D microenvironment to control the quality of the loaded cells before transplantations; (iii) facilitating the fast mechanical recovery of the damaged hydrogels, keeping intrinsic functionalities and expanding the service life of implanted cell-loaded hydrogels^26^,^27^,^28^.

So far, few self-healing hydrogels have been developed as injectable carriers for cell delivery. For example, Ma *et al.* presented a self-healing dextran-based hydrogel consisted of multiple-hydrogen-bond units (ureido-pyrimidinone, Upy) modified dextran chains, which can achieve rapid self-integration through the hydrogen bond between Upy units^29^. Two pieces of self-healed cell-loaded hydrogels which separately loaded with chondrocytes and bone marrow stem cells can form cartilage-bone tissue complex after subcutaneous implantation. Liu and coauthors synthesized self-healing injectable hydrogel via dynamic imine bonds between multiple aldehyde groups on chondroitin sulfate and N-succinyl-chitosan. The HeLa cells encapsulated into the hydrogel remain viable and metabolically active^30^. However, achieving the self-healing and injectable capabilities for NSC delivery in the treatment of neurological lesions still remains sparse. To this end, self-healing injectable hydrogels should possess the biomechanical property similar to that of native brain tissues, and should also provide a suitable microenvironment for the proliferation and neuronal differentiation of NSCs.

To address the emerging need for self-healing injectable hydrogels with improved functionality for the delivery of NSCs, we had developed a polysaccharide-based self-healing injectable hydrogel system mimicking the biomechanical properties of brain tissues. The polysaccharide networks of the self-healing injectable hydrogels, denoted as CEC-l-OSA hydrogel (“l” means “linked-by”), are cross-linked by the reversible imine bonds formed from a Schiff base reaction of the amino groups on N-carboxyethyl chitosan (CEC) and the aldehyde groups on oxidized sodium alginate (OSA). The chitosan and sodium alginate are chosen as the backbones of the polymer networks due to cytocompatibility, water solubility, low-cost, and abundant in nature^31^. Especially, the amino groups on chitosan promote nerve regeneration^32^,^33^,^34^,^35^. The imine bonds via Schiff reaction are attributed to the family of dynamic covalent bonds which can establish an intrinsic dynamic equilibrium of bond association and dissociation in hydrogel networks, offering self-healing capability to the CEC-l-OSA hydrogels. The self-healing injectable CEC-l-OSA hydrogels with the stiffness similar to nature brain tissues (100~1000 Pa)^36^,^37^ can be formed *in situ* via dynamic imine bonds, through facilely mixing the solution of CEC and OSA under physiological environment. Moreover, the NSCs loaded inside the self-healing injectable hydrogels maintain the normal functions of proliferation and differentiation, hold promises for the transplantation of NSCs and the treatment of neurological diseases.

## Results and Discussion

**Figure 1:**
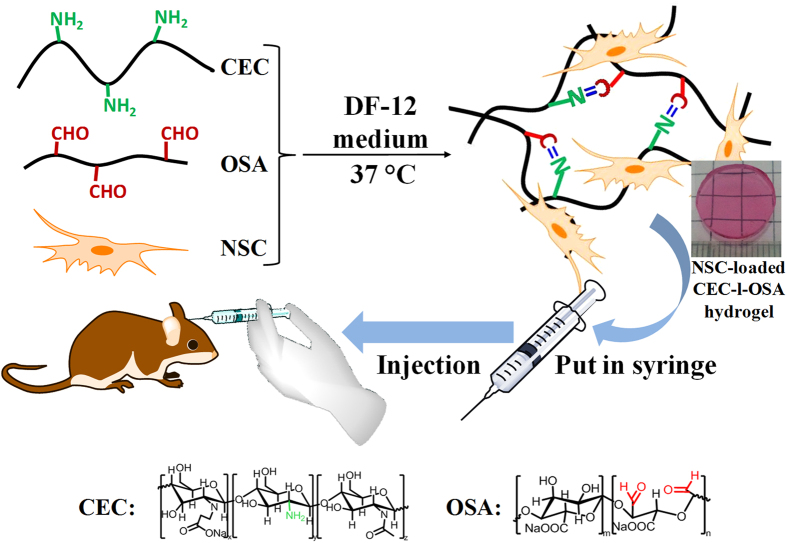
The transplantation strategy of injectable NSC-loaded, self-healing CEC-l-OSA hydrogels. CEC and OSA are dissolved into DF-12 medium, respectively, and the NSCs are suspended in the OSA medium. After mixing the DF-12 medium containing CEC and the cell suspension containing OSA, NSC-loaded CEC-l-OSA hydrogels can be obtained via Schiff base reaction. Subsequently, the needle of the syringe is inserted into a lesion cavity, and the cell-loaded hydrogel fragments will be injected into the lesion cavity for NSCs transplantation. (The drawings of the rat, the hand and the two syringes in the figure are obtained from the clip art library of ChemBioDraw Ultra 12.0).

The transplantation strategy of NSCs loaded CEC-l-OSA hydrogel is shown in Fig. 1. Briefly, cell-loaded CEC-l-OSA hydrogels were prepared in advance before the injection. Firstly, CEC and OSA were dissolved into DMEM-12 (DF-12) culture medium separately, and the NSCs were suspended in the OSA component. After mixing the CEC component and the OSA component with cells, NSC-loaded CEC-l-OSA hydrogels can be obtained via Schiff base reactions. To provide a microenvironment with mechanical properties to match that of brain tissues, as well as facilitating NSC proliferation and differentiation, the shear modulus of CEC-l-OSA hydrogels was adjusted to the order of 100~1000 Pa. The NSC-loaded CEC-l-OSA hydrogels exhibited excellent self-healing and injectable properties under physiological conditions. The cell loaded hydrogels were squeezed through a syringe to fill the lesion cavity. Subsequently, the cell-loaded fragments of hydrogel could self-heal into a whole at the target site, which would fill up the irregular shapes of lesion cavities. The detailed information of CEC-l-OSA hydrogel preparation, self-healing behaviors, mechanical properties, as well as proliferation and differentiation of NSCs inside the CEC-l-OSA hydrogels will be discussed here.

### Preparation of CEC-l-OSA hydrogel

Chitosan was modified with acrylic acid through Michael’s reaction to enhance its water solubility under physiological conditions, and the sodium alginate was periodate-oxidized to OSA with aldehyde groups^19^. The synthesis of CEC and OSA are well established, simple and green. It is an organic-solvent-free reaction and only water is used in the processes of modification. Moreover, the only byproduct of the dynamic Schiff crosslinking reaction is water and the cytotoxicity of the hydorgel is minimal. (Supplementary Fig. S1). In order to meet the actual demand of cell cultivation and transplantation, the CEC-l-OSA hydrogels were prepared in DF-12 medium of NSCs under physiological conditions. The CEC-l-OSA hydrogels can be facilely prepared *in situ* by mixing CEC DF-12 solution and OSA DF-12 solution via Schiff base reaction. The polysaccharide chains are cross-linked by dynamic imine bonds among the reactive groups modified on the polymer chains, i.e., amino groups on CEC and the aldehyde groups on OSA. A series of CEC-l-OSA hydrogels were obtained by mixing the OSA solution of a fixed concentration (*C*_o_ = 0.1 g/mL) with different concentration of the CEC solution (*C*_c_ = 0.01~0.03 g/mL). The molar ratio of the reactive groups was kept at 1 (M-NH_2_: M-CHO = 1) to ensure the theoretically complete cross-linking of the amino groups and aldehyde groups in two components.

**Figure 2:**
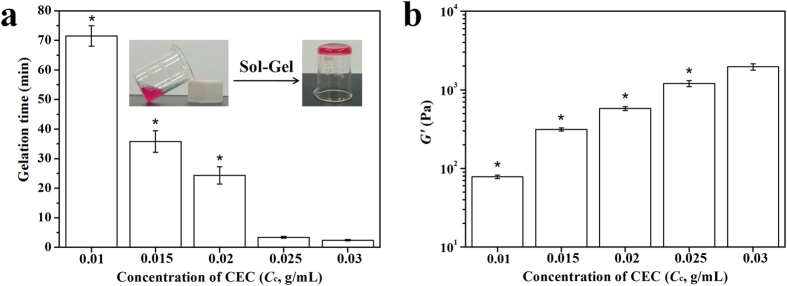
The gelation time and storage moduli (*G*′) of the CEC-l-OSA hydrogels with various CEC solutions (*C*_c_ = 0.01~0.03 g/mL) under physiological conditions. (**a**) The gelation time of the CEC-l-OSA hydrogels. (**b**) The *G*′ of the CEC-l-OSA hydrogels. The data are extracted from the plateaus of variation of *G*′ versus angular frequency (1 to 10 rad/s). *Symbol indicated the significant differences (p < 0.05) between samples with C_c_ = 0.03 g/mL and samples at other CEC concentrations. Error bars represent standard deviations (n = 3).

The vial inversion method was used to determine gelation times of the CEC-l-OSA hydrogels with various CEC concentrations (*C*_c_). At 37 °C, the flowability of the CEC and OSA mixtures dramatically decreased with mixing time. The CEC-l-OSA hydrogels were formed via the dynamic covalent imine cross-links. The gelation time of the CEC-l-OSA hydrogels was dependent on CEC concentration, which took the longest time of 71.5 ± 3.5 min for the lowest *C*_c_ of 0.01 g/mL. When gradually increasing the *C*_c_ from 0.01 to 0.03 g/mL at intervals of 0.005 g/mL, the gelation was accelerated and the gelling time sharply decreased to 35.8 ± 3.6, 24.4 ± 2.9, 3.4 ± 0.3 and 2.4 ± 0.2 min for *C*_c_ = 0.015, 0.02, 0.025 and 0.03 g/mL, respectively (Fig. 2a). These results indicate that the higher the concentration of CEC and the reactive groups of amino and aldehyde in the networks, the faster gelation of the hydrogel. Whereas, for the samples with low CEC concentrations (*C*_c_ < 0.01 g/mL), hydrogel formation was not observed, indicating that as the concentrations of the polymers and reactants are too low to meet the minimum requirements for gelation. On the other hand, the samples with *C*_c_ > 0.03 g/mL failed to form homogeneous hydrogels due to the high viscosities of the polymer solutions preventing homogenous mixing.

To study the effect of CEC concentration on the mechanical properties of the CEC-l-OSA hydrogels, we performed the rheological test of the hydrogels with various *C*_c_ (Fig. 2b). The storage modulus (*G*′) rose from 77.9 ± 3.9 Pa to 1961 ± 178 Pa when *C*_c_ was increased from 0.01 to 0.03 g/mL. The higher concentrations of reactive groups as well as the increased amount of CEC, contribute to a significant increase in storage modulus. It is well-known that the *G*′ of normal neural tissues are in the range of 10^2^~10^3^ Pa^38^,^39^, which can be fully covered with the adjustable *G*′ of CEC-l-OSA hydrogels through controlling the concentration of CEC. This indicates that the CEC-l-OSA hydrogels can be potentially used as biomaterials for neural tissue engineering.

The CEC-l-OSA hydrogels are degradable through hydrolysis. The degradation behaviors of the CEC-l-OSA hydrogels with the various concentrations of CEC were investigated in DF-12 medium under 37 °C (Supplementary Fig. S4), The hydrogels swelled in the DF-12 medium in the first few days, and then degraded by 3 to 14 days along with the *C*_c_ varying from 0.01 to 0.03 g/mL. Besides the hydrolysis, the polypeptides (such as growth factors and serum with amino groups) contained in the DF-12 medium may also contribute to the degradation of the polymer networks.

### Self-healing behavior and mechanical recovery of the injectable CEC-l-OSA hydrogel

**Figure 3:**
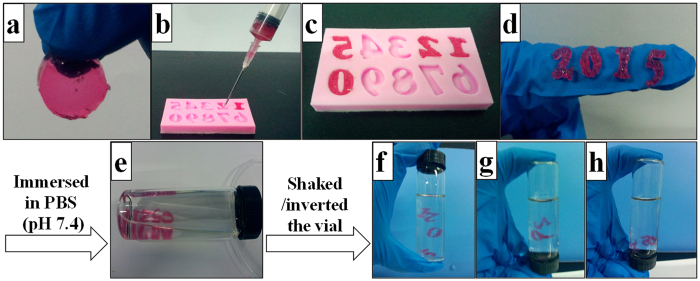
The injectable performance and self-healing capability of one CEC-l-OSA hydrogel (*C*_c_ = 0.02 g/mL). (**a**) A disc-shaped hydrogel is formed after mixing CEC and OSA. (**b**) Gelled material was loaded into a syringe and squeezed out into the “2”, “0”, “1”, “5” spots in a number mold. (**c**) The gel fragments were self-healed into continuous “2015” structures after 5 minute-culture at 37 °C without any external intervention. (**d**–**h**) The self-healed structures were stable in PBS (pH 7.4) under shaking and inverting motions.

Considering the dynamic imine cross-links existed in the hydrogel networks and the low storage modulus, the CEC-l-OSA hydrogels can be injected from syringes through needles into the lesion sites. The hydrogels possess the self-healing ability after injection. To test the injectability and self-healing ability, CEC-l-OSA hydrogels with CEC concentration at 0.02 g/mL and *G*′ at 577 ± 31 Pa were tested as a model hydrogel. A hydrogel disc was put into a 21-gauge syringe and extruded through a needle into number molds with the shape of “2”, “0”, “1”, “5” (Fig. 3a,b). The number molds filled hydrogel fragments were left at 37 °C for 5 min without any external interventions, the hydrogel fragments self-healed via dynamic imine bonds and turned into dense hydrogels with the shape of the numbers (Fig. 3c). The hydrogels in number shapes were stable when exposed to gentle shaking and inverting of the vials filled with phosphate buffered saline (PBS) (pH = 7.4) (Fig. 3d–h). These tests demonstrated good injectability and the excellent self-healing capability of the CEC-l-OSA hydrogels when exposed to the physiological saline.

**Figure 4:**
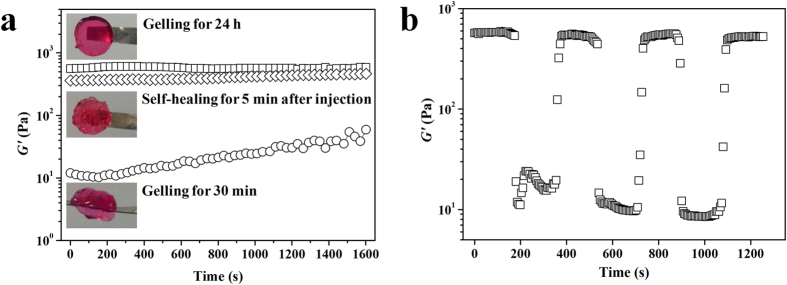
The rheological measurement of self-healing CEC-l-OSA hydrogel. (**a**) The *G*′ of the CEC-l-OSA hydrogels after setting for 24 h (□) and 30 min (◊), as well as self-healing for 5 min after injection through a needle (○). (**b**) The *G*′ of the CEC-l-OSA hydrogel from alternate step strain sweep with small strain (*γ* = 0.1%) for 100 s and following large strain (*γ* = 1000%) for 200 s at a fixed angular frequency (10 rad/s) at 37 °C.

To assess the mechanical properties of the self-healing CEC-l-OSA hydrogels after injection, we performed the rheological measurements of the samples prepared in DF-12 culture medium (*C*_c_ = 0.02 g/mL) under 3 different conditions (Supplementary Fig. S5). One was a self-healing samples, i.e., injecting disk-shaped hydrogels into a circular mold via a syringe with a needle, then the fragments of the hydrogels were set aside for 5 min (denoted as *H*_S_). The second sets were samples of freshly gelled, i.e., directly mixing CEC DF-12 solution and OSA DF-12 solution into a circular mold. After gelation for 25 minutes, the samples were set for 5 more minutes before the rheological test (denoted as *H*_*T*_). The third sets of samples (control) were prepared at the same condition as the sample set # 2, but set aside for 24 h before the rheological test (denoted as *H*_C_). When sweeping with time, the *G*′ of the *H*_S_ samples immediately reached up to 365 Pa at initial time, which is close to that of the *H*_C_ sample (577 Pa). However, at start time, the *G*′ of the *H*_T_ sample was as low as 12 Pa which is 30-48 times lower than that of the *H*_S_ and *H*_C_ samples. Moreover, the *G*′ slowly rose with sweeping time and only reached 59 Pa after 1600 s (Fig. 4a). It indicates that the mechanical modulus of the CEC-l-OSA hydrogels can be built up more quickly by adopting the self-healing injection manner. Whereas, seriously delaying in the increment of the storage modulus over a long period of time is exhibited when the sample is freshly prepared from two components as in conventional injection with two-syringe based mixing at the time of injection. CEC-1-OSA hydrogels may have a potential risk of hydrogel displacement and cell loss during the injection when using conventional mixing approach during the transplantation. The results indicate that the injectable self-healing CEC-l-OSA hydrogels support fast mechanical recovery, facilitate to keep intrinsic functionalities, guard against cargo loss, as well as expand the service life of injected hydrogels.

To further confirm the self-healing behaviors of the CEC-l-OSA hydrogel after injection, the continuous step-strain measurements were conducted to test the rheological recovery behaviors of the *H*_S_ hydrogels (Fig. 4b). Briefly, the oscillatory shear strain of 0.1% and 1000% were alternately loaded on the disk-shaped CEC-l-OSA hydrogels at a fixed frequency of 0.1 rad/s under 37 °C, and every strain were kept for 200 s. As expected, the *G*′ of the hydrogel could be repeatedly recovered to the original value after being broken down under large amplitude oscillatory. The results indicate that the CEC-l-OSA hydrogels with excellent self-healing performances can effectively repair the possibly damages due to mechanical interruption of the hydrogel structures, improve the functionalities and security level of cell transplantation with hydrogels as carriers.

### Cytocompatibility, proliferation and differentiation of NSCs encapsulated in CEC-l-OSA hydrogel

**Figure 5:**
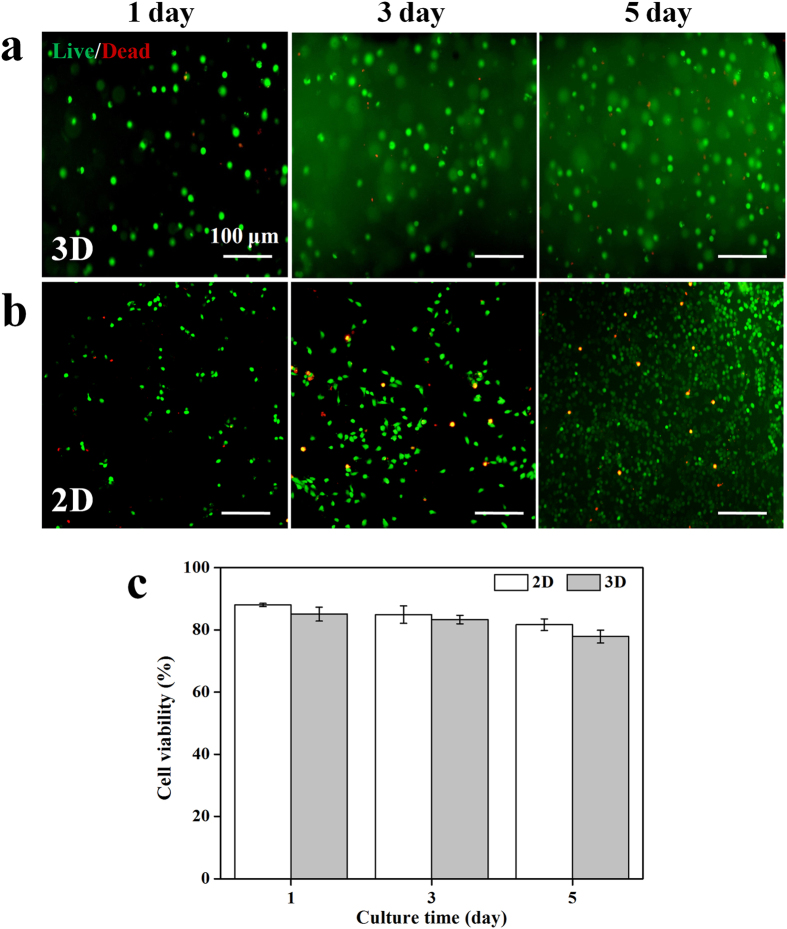
The cell viability of 3D and 2D cultures of NSCs. (**a**) Live/Dead staining of NSCs loaded inside the 3D CEC-l-OSA hydrogels (*C*_c_ = 0.02 g/mL) for 1, 3 and 5 days. (**b**) Live/Dead staining of NSCs on the surface of tissue culture plastics for 1, 3 and 5 days. Live: green, dead: red. (**c**) The cell viability of NSCs under 2D and 3D conditions of different culture times of 1, 3 and 5 days. There is no significant difference on the cell viability between 3D and 2D groups. Error bars represent standard deviations (n = 3).

To confirm the possibility of using the self-healing injectable hydrogels for nerve repair, the cytocompatibility, proliferation and differentiation of NSCs loaded inside the CEC-l-OSA hydrogels were examined and analyzed. The cytocompatibility of the model self-healing CEC-l-OSA hydrogels with *C*_c_ at 0.02 g/mL, was confirmed through Live/Dead test of NSCs loaded inside 3D hydrogels. The cell-loaded hydrogels can be facilely prepared by mixing NSCs suspension with CEC and OSA dissolved in DF-12 medium under physiological conditions (Fig. 5a). In contrast, the 2D culture of NSCs on the surface of commercial cell culture plates made of polystyrenes coated with polylysine and polyornithine/laminin were used as a 2D control (Fig. 5b). The cells stained in green are live cells, whereas cells stained in red are dead cells. The NSCs were uniformly dispersed into CEC-l-OSA hydrogels and exhibited *c*.*a*. 90% cell viability after cultivation for 1, 3 and 5 days, which is similar to the control samples (Fig. 5c). These results demonstrate that the CEC-l-OSA hydrogel exhibits excellent cytocompatibility for NSCs.

**Figure 6:**
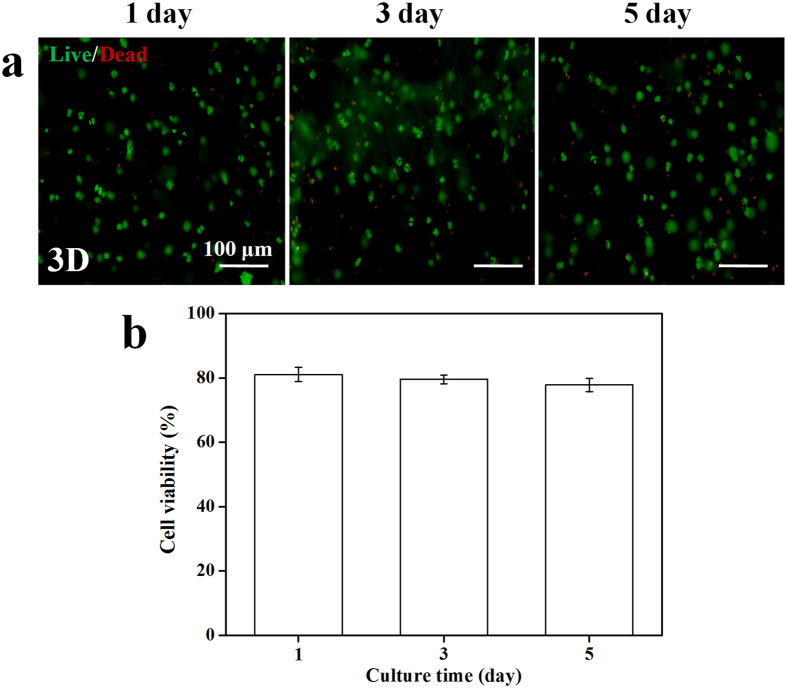
The cell viability of injected NSCs encapsulated in CEC-l-OSA hydrogels (*C*_c_ = 0.02 g/mL). (**a**) Live/Dead staining of the encapsulated NSCs after injection for 1, 3 and 5 day. Live: green, dead: red. (**b**) The cell viability versus different culture times. Error bars represent standard deviations (n = 3).

To further evaluate the effects of injection through a needle on the cell viability, a piece of as-prepared NSC-loaded CEC-l-OSA hydrogel was push through a 21-gauge needle into a 24-well tissue culture plate (Fig. 6a). The extruded hydrogel fragments self-healed into one single piece hydrogel in each well after few minutes, and the cells loaded inside the self-healed hydrogels were continuously cultured. Although the extrusion pressure from the syringe may cause cellular damage, the cell viability of NSCs loaded inside the self-healed CEC-l-OSA hydrogel was ∼80% after cultivation for 1, 3 and 5 days (Fig. 6b).

**Figure 7:**
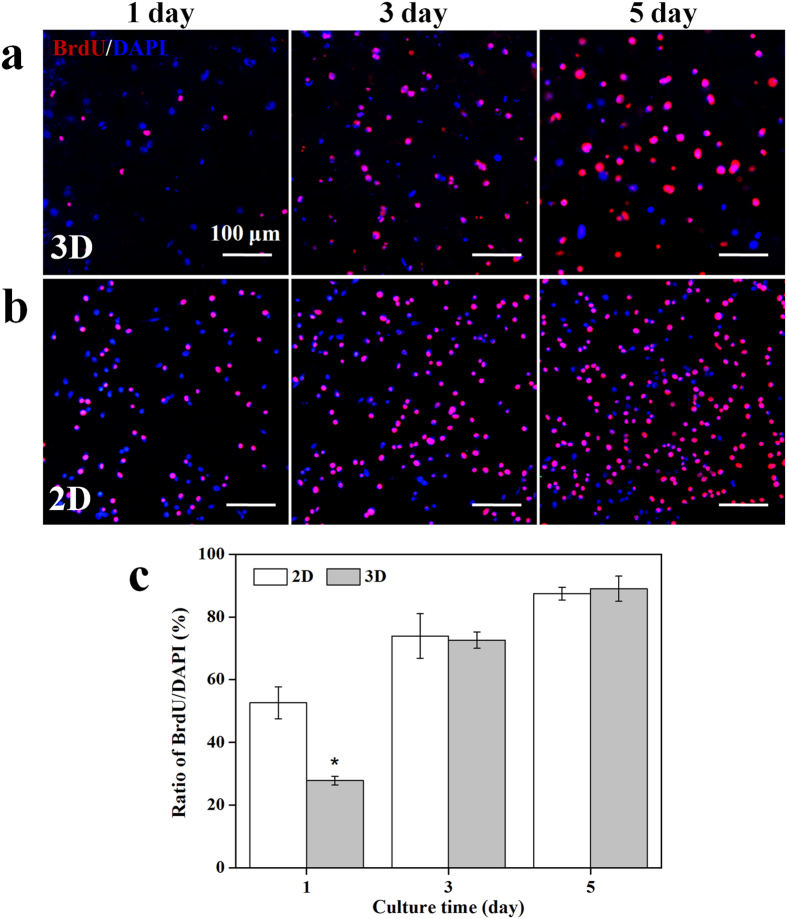
The proliferation of NSCs under 3D and 2D cultures. (**a**) The proliferation of NSCs loaded inside the CEC-l-OSA hydrogels (*C*_c_ = 0.02 g/mL) for 1, 3 and 5 days, stained with BrdU/DAPI. (**b**) The proliferation of NSCs cultured on the surface of tissue culture plates for 1, 3 and 5 days, stained with BrdU/DAPI. All cell nuclei were stained with DAPI in blue, and the dividing cells were immunostained with anti-BrdU antibody in red. The cells with violet color derived from the overlay of blue and red color are the divided cells. (**c**) The percentage of proliferating cells, i.e., the ratio of BrdU positive cells to DAPI stained ones, at different culture times of 1, 3 and 5 days. *Symbol indicated the significant differences (p < 0.05) between 2D and 3D cultures. Error bars represent standard deviations (n = 3).

To analyze the proliferation of NSCs loaded inside the CEC-l-OSA hydrogels, BrdU/DAPI staining was used for evaluating cell proliferation *in vitro*. All the cell nuclei stained with DAPI are in blue, and the dividing cells immunostained with anti-BrdU antibody are in red. Thus, the cells with violet color are from the overlay of blue and red colors are the newly divided cells (Fig. 7a). The 2D cultivation of NSCs on commercial cell culture plates coated with polylysine and polyornithine/laminin was set as control (Fig. 7b). The results of BrdU/DAPI staining showed that the number of NSCs loaded inside the CEC-l-OSA hydrogels increased with culture time (1, 3 and 5 day) (Fig. 7a). When being cultivated for 1 day, the proliferation rate of the cells encapsulated in the CEC-l-OSA hydrogel was only 27.8 ± 1.4%, which was significantly lower than that of the 2D control group (52.6 ± 5.1%). After cultivation of 3 and 5 days, the proliferation rate of the cells encapsulated in the CEC-l-OSA hydrogel was increased to 72.6 ± 2.6% and 89.0 ± 4.0, respectively, which was similar to that of the 2D control group (79.3 ± 7.1% for 3 days and 87.4 ± 2.0% for 5 days) (Fig. 7c). It is considered that the NSCs may need appropriate time to adapt the 3D cultivation microenvironment on the initial day. After the adaptive stage, the NSCs can proliferate with culture time. These results illustrate that the CEC-l-OSA hydrogels are cytocompatible and favorable supporting scaffolds for the proliferation of NSCs in 3D microenvironments.

**Figure 8:**
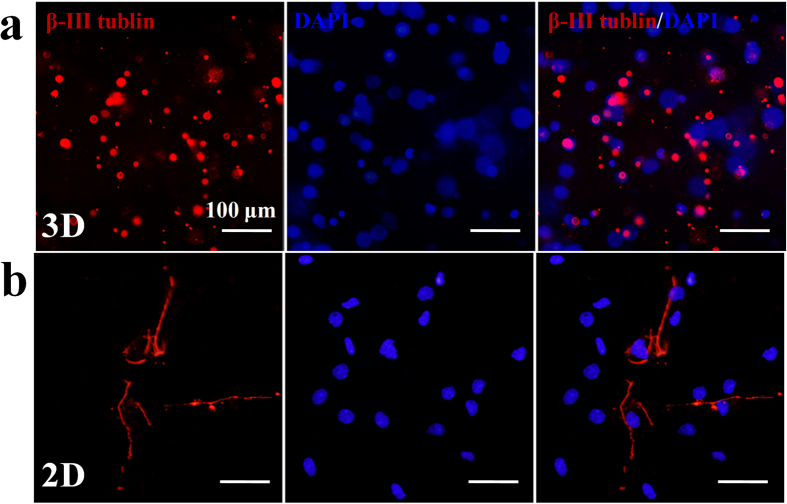
The neuronal differentiation of 3D and 2D cultured NSCs. (**a**) The expression of β-III tubulin marker of the NSCs loaded inside the CEC-l-OSA hydrogels (*C*_c_ = 0.02 g/mL) for 9 days. (**b**) The expression of β-III tubulin marker of the NSCs cultured on 2D cell culture plates for 9 days. All the cell nuclei stained with DAPI are blue color, and the cells immunostained with β-III tubulin marker are red color.

**Figure 9:**
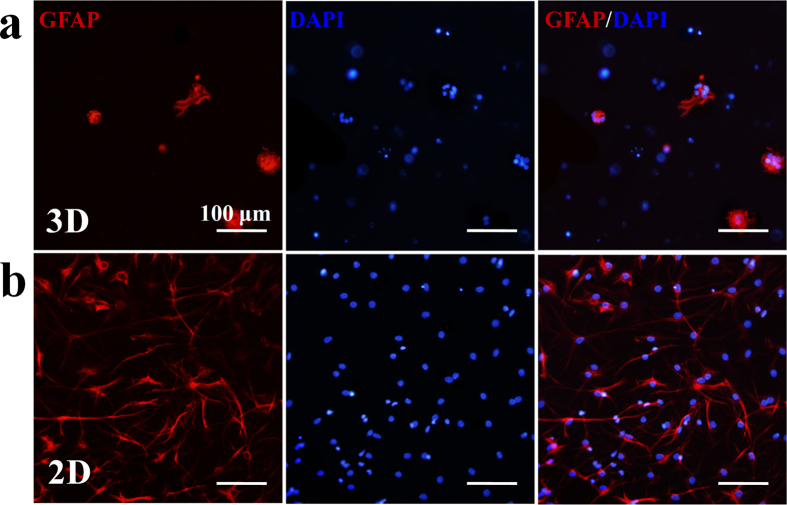
The glial differentiation of 3D and 2D cultured NSCs. (**a**) The expression of GFAP marker of the NSCs loaded inside CEC-l-OSA hydrogels (*C*_c_ = 0.02 g/mL) for 9 days. (**b**) The expression of GFAP marker of the NSCs cultured on 2D cell culture plate surfaces for 9 days. All the cell nuclei were stained with DAPI in blue, and the cells were immunostained with GFAP marker in red.

The CEC-l-OSA hydrogels also facilitate neuronal differentiation of NSCs. The neural stem cell marker, nestin, was demonstrated positively both for 3D hydrogel group and 2D control group on the first day (Supplementary Fig. S6). After replacing the differential medium and continuously cultured for 9 days, the 3D cultured cells inside the CEC-l-OSA hydrogels express much more neuronal marker, β-III tubulin, than the 2D control group (Fig. 8a,b). In contrast, the expression level of the glial marker GFAP in CEC-l-OSA hydrogels displayed the opposite results (Fig. 9a,b). The differentiated neuronal cells inside the CEC-l-OSA hydrogels were 38% higher than the 2D cultured group but the glial cells were 51% less than the control (Supplementary Fig. S7). This consequence may be not only attributed to the suitable stiffness of the CEC-l-OSA hydrogels (~500 Pa), but also the chemical structure of the hydrogel. The dynamic cross-links of imine bonds containing dissociated amine groups in the CEC-l-OSA hydrogel networks can induce and promote the neuronal differentiation^32^,^33^,^34^,^35^. The differentiated neurons cultured on commercial cell culture plates extended long neurites (Fig. 8b). However, the morphology of neurons inside the hydrogel was still rounded, which should be a result of the limited growth of neurites in the restrictive 3D polymer networks of the hydrogel. We also injected the NSC-loaded CEC-l-OSA hydrogels in mice with focal cerebral ischemia model (Supplementary Fig. S8). The NSC-loaded hydrogels fully filled up the lesion site and the NSCs were observed uniformly distributed in the hydrogels.

## Conclusion

In summary, we had developed a neurocompatible, injectable, and self-healing CEC-l-OSA hydrogel system, which was obtained by a Schiff reaction between polysaccharide CEC and OSA. The resultant dynamic imine bonds existed in the hydrogel networks impart the self-healing capability to the hydrogel under physiological conditions, which is confirmed by the injectable performance and self-healing capability based on rheological recovery tests and macroscopic observations. The biocompatible CEC-l-OSA hydrogels with the similar stiffness of nature brain tissues support the proliferation of NSCs loaded inside the 3D hydrogels. CEC-l-OSA hydrogels also favor neuronal differentiation of NSCs loaded inside the gels. The preparation of the CEC-l-OSA hydrogels is simple, scalable, low-cost and environment friendly. This investigation opens the door to polysaccharide-based self-healing hydrogels in the field of neural stem cell transplantation.

## Methods

### Materials

Sodium alginate (>350 mpa.s), acrylic acid, and sodium periodate were purchased from Alfa Aesar. Chitosan (degree of deacetylation 86%, Mw = 200,000 Da) was ordered from Tokyo Kasei Kogyo Co., Ltd. All other chemicals were analytical grade and used without further purification.

### Experimental procedures

#### Synthesis of N-carboxyethyl chitosan (CEC)

N-carboxyethyl chitosan was synthesized by our reported method^19^. Briefly, chitosan (0.5 g, 3.1 mmol) was dissolved in 25 mL distilled water containing acrylic acid (0.73 mL, 10.65 mmol), and the mixture was magnetically stirred under 50 °C for 3 days. Then the pH of the solution was adjusted to 10–12 by adding 1 mol/L NaOH solution dropwise. Thereafter, the solution was dialyzed (MWCO 8000) against distilled water for 3 days with repeated change of water, followed by freeze-dried to obtain the pure CEC powder. Typical yield of the products was ∼75%. The degree of substitution was around 38%, which was determined according to the ^1^HNMR spectra by comparing the peak area of the acetamide methyl protons (δ = 1.94) in chitosan and the methylene protons (δ = 2.83) of acrylic acid in CEC (^1^H NMR (400 MHz, D_2_O, δ): 1.94 (s, 3 H, COCH_3_), 2.83 (s, 2 H, CH_2_CO_2_Na), 3.30~4.87 (m, glucosamine)).

#### Synthesis of oxidized sodium alginate (OSA)

The synthesis of OSA was based on the previous method with a slight modification^19^. Sodium alginate (1.0 g, 5 mmol) was dissolved in 100 mL distilled water, then sodium periodate (0.54 g, 2.5 mmol) was added, and the solution was magnetically stirred in the dark at 25 °C for 5 h. The reaction was terminated by adding ethylene glycol (1.5 mL) and stirred for additional 1 h. After the reaction, the mixture was dialyzed (MWCO 3000) against distilled water for 3 days with repeated change of water, followed by lyophilizing to obtain the products of OSA. Typical yield of the products was ∼80%.

#### Determination of the degree of oxidation (DO)

The DO was evaluated by the iodometry through determining the concentration of unconsumed periodate after the oxidized reaction. The 20 wt% potassium iodide solution (2 mL) was added to the reaction mixture (5 mL) after it was neutralized by adding 10 mL of 10 wt% sodium bicarbonate solution. The reaction was stirred in the dark at 25 °C for 30 min, and then the liberated iodine was titrated with standardized sodium thiosulphate solution (0.01 mol/L) using starch (1 wt%) as the indicator. The value of DO is 54%, which is averaged from triplicate oxidation experiments.

#### Preparation of CEC-l-OSA hydrogel

The DMEM/F-12 (DF-12) medium solution of OSA (*C*_o_ = 0.1 g/mL) were mixed with DF-12 solutions containing different concentrations of CEC (*C*_c_ = 0.01, 0.015, 0.02, 0.025 and 0.03 g/mL) at a fixed molar ratios (M-NH_2_: M-CHO = 1) under 37 °C. The solution was mixed uniformly by vortex and eventually homogeneous hydrogels were obtained.

#### Measurement of gelation time of CEC-l-OSA hydrogels

Vial inversion method was applied to determine the gelation time. When no flow was observed and the different concentrations of CEC (*C*_c_ = 0.01, 0.015, 0.02, 0.025 and 0.03 g/mL) with OSA (*C*_o_ = 0.1 g/mL) mixtures would be inversed at 37 °C, they were regarded as hydrogels. All the gelling times were measured in triplicate for each group.

#### Rheological measurements

*1*) The storage moduli (*G*′) of CEC-l-OSA hydrogel discs (15 mm in diameter) with various *C*_c_ (0.01, 0.015, 0.02, 0.025 and 0.03 g/mL) and fixed *C*_o_ = 0.1 g/mL were tested with a rheometer equipped with parallel dentate anti-skid plates (both top and bottom plates are 15 mm in diameter) at 37 °C. Under a fixed strain of 0.1%, the angular frequency was swept from 0.1 rad s^−1^ to 100 rad s^−1^. The *G*′ of CEC-l-OSA hydrogel discs (15 mm in diameter) with various *C*_o_ (0.05, 0.1 and 0.2 g/mL) and fixed *C*_c_ = 0.02 g/mL were also tested by rheometer under same conditions (Supplementary Fig. S2). *2*) The *G*′ of the CEC-l-OSA hydrogels (*C*_c_ = 0.02 g/mL) with gelation time of 5 min and 24 h as well as the self-healing hydrogel with healing time of 5 min after injection, were tested at 37 °C under a fixed strain of 0.1% and angular frequency of 10 rad s^−1^. *3*) The alternate step strain sweep of CEC-l-OSA hydrogel discs (15 mm in diameter) with *C*_c_ = 0.02 g/mL was measured at a fixed angular frequency (10 rad s^−1^) at 37 °C. Amplitude oscillatory strains were switched from small strain (*γ* = 0.1%) to subsequent large strain (*γ* = 1000%) with 200 s for every strain interval.

#### Measurement of the degradation rates of CEC-l-OSA hydrogels

The degradation of CEC-l-OSA hydrogels (*C*_c_ = 0.01, 0.015, 0.02, 0.025 and 0.03 g/mL) was determined through weighing the samples at different time points. Hydrogels (*W*_0_) were immersed in microtubes with 500 μL of DF-12 medium at 37 °C. The medium was removed from each sample at pre-determined time points. The weight of each samples (*W*_t_) were measured. Fresh medium were added into the microtubes after each weighing. The degradation ratio was then calculated by *W*_t_/*W*_0_.

#### Self-healing test of the CEC-l-OSA hydrogels

Disc-shaped CEC-l-OSA hydrogels (*C*_c_ = 0.02 g/mL) were put into syringes and extruded through 21-gauge needles into number molds of “2”, “0”, “1”, “5” and cultured for 5 min at 37 °C without any external intervention. The newly formed hydrogel numbers “2”, “0”, “1”, “5” were taken out from the mold and immersed in PBS (pH 7.4). Their stability was observed by gently shaking and inverting the vials.

#### Neural stem cells (NSCs) isolation and culture

*1*) NSCs were isolated from cerebral cortex of fetal rats at E14. The cells were expanded as neurospheres in serum-free DF-12 medium supplemented with 100 mg/mL penicillin-streptomycin, 20 ng/mL epidermal growth factor (EGF), 10 ng/mL basic fibroblast growth factor (bFGF), B27 supplement and N2 supplement in incubator for 3–5 days. After trypsin incubation, cells were subcultured on poly-lysine and poly-ornithine/laminin-coated commercial tissue culture plates. *2*) As for differentiation study, NSCs dissociated from neurospheres were seeded on culture plates as above and cultured in differentiation medium consisting of DF-12 medium, penicillin-streptomycin, B27 supplement, N2 supplement and fetal bovine serum (FBS) for 9 days. The medium was exchanged every other day.

#### NSC loading into CEC-l-OSA hydrogels

For the tests of cell viability and proliferation, NSCs were loaded inside CEC-l-OSA hydrogels in a density of 1 × 10^6^ cells/mL. For the differentiation study, the density was 1 × 10^5^ cells/mL. Briefly, CEC (*C*_c_ = 0.02 g/mL) and OSA (*C*_o_ = 0.1 g/mL) were dissolved in DF-12 medium, respectively. After trypsin digestion and centrifugation of NSCs, NSCs were re-suspended in 900 μL CEC DF-12 solution followed by mixing with 100 μL OSA DF-12 solution. The mixture was rapidly plated in 24 well-plates within 30 s to form gel samples in each well. In order to collect these gel samples, we pre-positioned one cover slip in each well. The control group was the culture of NSCs on commercial cell culture plates. The samples were placed in a CO_2_ incubator at 37 °C and 50% of the medium was changed every 3 days. For the proliferation study, bromodeoxyuridine (BrdU) was added to each well. These NSC-loaded CEC-l-OSA hydrogels were collected and processed by the following evaluations. *1*) Cell viability was determined by a live/dead assay after culturing for 1, 3 and 5 days. *2*) Cell proliferation test was assessed by immunofluorescent staining after culturing for 1, 3 and 5 days. *3*) Cell differentiation was examined by immunofluorescent staining after culturing for 9 days.

#### Cell viability test

To investigate the viability of NSCs, a live/dead assay was performed. Briefly, the NSCs loaded CEC-l-OSA hydrogels were rinsed with 0.01 mol/L PBS. In dark condition, 300–500 μL of stain solution (prepared by 4 mmol/L ethidium homodimer-1 and 2 mmol/L calcein AM in 0.01 mol/L PBS) was added into each well and the samples were incubated for 3 min at room temperature. The stain solution was discarded and the samples were washed with 1X PBS twice. Then they were fixed with 4% paraformaldehyde for 30 min and washed with 1X PBS again. 50% glycerol was added on the samples as mounting medium before fluorescence microscope observation and analysis.

#### Immunostaining

Immunostaining was performed to identify NSCs, investigate proliferation, and detect differentiation of cells seeded inside the CEC-l-OSA hydrogels. *1*) For the proliferation detection, cells were firstly fixed with 4% paraformaldehyde for 20 min, then incubated with 2 mol/L HCl solution (in order to open the double-stranded DNA of cells) for 1 h, and neutralized with 0.1 mol/L boric acid solution. After that, the cells were permeabilized with 0.3% Triton X-100 for 30 min, and blocked in 2% goat serum for 1 h at room temperature. Then they were incubated with primary antibody, mouse monoclonal anti-BrdU (1:200 dilution) for 2 h at room temperature and at 4 °C overnight. Thereafter, the primary antibody solution was discarded and samples were washed with 1X PBS for three times. The secondary antibody, anti-mouse Cy3 secondary antibody (1:2000 dilution), was added and incubated for 2 h at room temperature. DAPI (1:1000 dilution) solution was used to stain nuclei for 5 min at room temperature. *2*) For identify NSCs, cells were fixed with 4% paraformaldehyde for 20 min, permeabilized with 0.3% Triton X-100 for 30 min, and blocked in 2% goat serum for 1 h in room temperature. Thereafter, samples were incubated with primary antibody, mouse monoclonal anti-nestin (1:200 dilution), for 2 h at room temperature and at 4 °C overnight. Then the primary antibody solution was discarded and the samples were washed with 1X PBS for three times. The secondary antibody, anti-mouse FITC secondary antibody (1:200 dilution), was added and incubated for 2 h at room temperature. DAPI (1:1000 dilution) solution was used to stain nuclei for 5 min at room temperature. *3*) For differentiation evaluation, cells were fixed with 4% paraformaldehyde for 20 min, permeabilized with 0.3% Triton X-100 for 30 min, and blocked in 2% goat serum for 1 h at room temperature. Thereafter, the samples were incubated with primary mouse monoclonal anti-β-tublin III (1:200 dilution), and rabbit monoclonal anti-GFAP (1:200 dilution) for 2 h at room temperature and at 4 °C overnight. Then the primary antibody solution was discarded and cells were washed with 1X PBS for three times. The following secondary antibodies including anti-mouse Cy3 (1:2000 dilution) and anti-goat TRITC (1:200 dilution) were then subsequently added, respectively, and incubated for 2 h at room temperature. DAPI (1:1000 dilution) solution was used to stain nuclei for 5 min at room temperature. Samples were examined under fluorescence microscope and quantitatively analyzed.

#### Cells and animal study

The rats were purchased from the Experiment Animal Center, Medical School of Xi’an Jiaotong University (Xi’an, China). The animals were housed and handled in strict accordance with the Guidelines of the Institutional and National Committees of Animal Use and Protection. The protocols were approved by the Committee on the Ethics of Animal Experiments of Xi’an Jiaotong University (Certificate No. 22-9601018). All efforts were made to minimize animals’ suffering and the numbers of animals used. All methods were performed in accordance with the relevant guidelines and regulations by including a statement in the Methods section to this effect.

## Additional Information

**How to cite this article**: Wei, Z. *et al.* Self-healing polysaccharide-based hydrogels as injectable carriers for neural stem cells. *Sci. Rep.* **6**, 37841; doi: 10.1038/srep37841 (2016).

**Publisher's note:** Springer Nature remains neutral with regard to jurisdictional claims in published maps and institutional affiliations.

## Supplementary Material

Supplementary Information
