# Supplementary material for: Self-healing polysaccharide-based hydrogels as injectable carriers for neural stem cells
Source: Sci Rep. 2016 Nov 29;6:37841. doi: 10.1038/srep37841 (PMC5126669; doi:10.1038/srep37841)
Supplement: Supplementary Information [file srep37841-s1.pdf]

Supplementary Information

Self-healing polysaccharide-based hydrogels as injectable carriers for neural stem cells

Zhao Wei [1,+], Jingyi Zhao [2,+], Yong Mei Chen [1,*], Pengbo Zhang [2,*], Qiqing Zhang[3,*]

[1] State Key Laboratory for Strength and Vibration of Mechanical Structures, International Center for Applied Mechanics and School of Aerospace, Collaborative Innovation Center of Suzhou Nano Science and Technology, Xi'an Jiaotong University, Xi'an 710049, China.

[2] Department of Anesthesiology, The Second Affiliated Hospital of Xi'an Jiaotong University, Xi'an 710003, China

[3]Institute of Biomedical and Pharmaceutical Technology, Fuzhou University, Fuzhou 350002, China, Fujian Guided Tissue Regeneration (GTR) Biotechnology Co., Ltd., Fuzhou 350108, China

Corresponding author. e-mail: chenym@mail.xjtu.edu.cn, zhpbo@163.com, zhangqiq@126.com

[+]These authors contributed equally to this work.

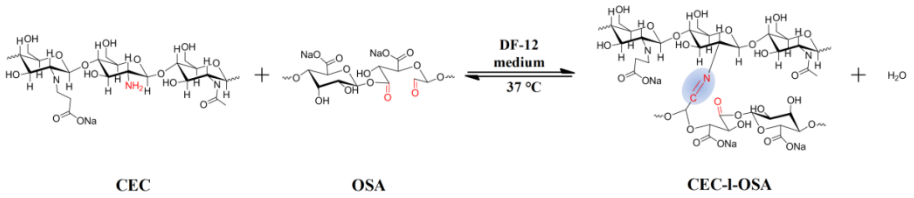

Figure S1 The dynamic schiff base reaction between amino groups on CEC and the aldehyde groups on OSA.

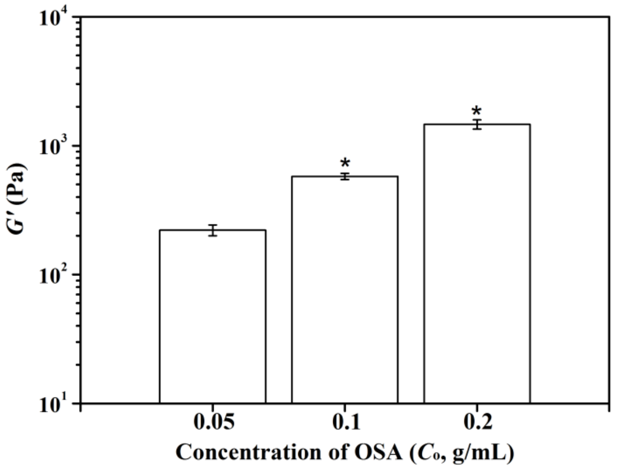

Figure S2 The G' of the CEC-l-OSA hydrogels with fixed CEC solution (C_c = 0.02 g/mL) and various OSA solutions (C_o = 0.05, 0.1 and 0.03 g/mL) under physiological conditions. The data are extracted from the plateaus of variation of G' versus angular frequency (1 to 10 rad/s). * symbol indicated the significant differences ($p < 0.05$) between samples with C_o = 0.05 g/mL and samples at other OSA concentrations. Error bars represent standard deviations (n = 3).

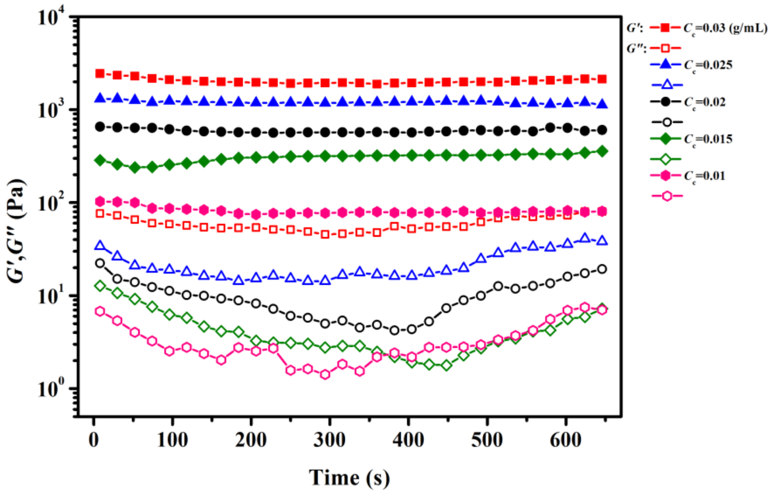

Figure S3 The *G*′ and *G*″ of the CEC-l-OSA hydrogels with various CEC concentration (C_c = 0.01~0.03 g/mL) by time sweep at angular frequency of 10 rad s^{-1} and a fixed strain (0.1 %).

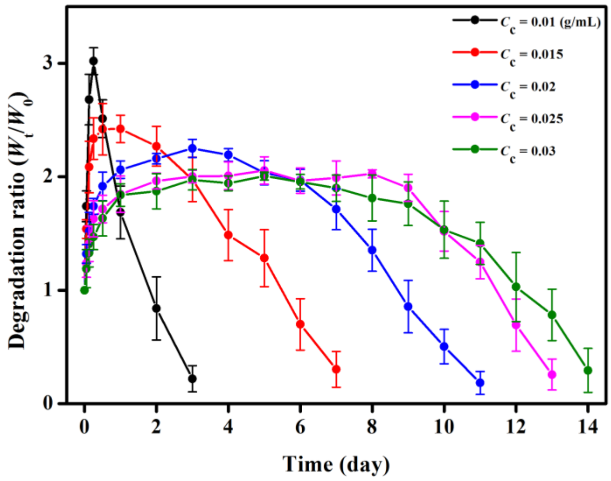

Figure S4 The degradation ratio of the CEC-l-OSA-l-ADH hydrogels with various CEC concentration (C_c = 0.01~0.03 g/mL) in DF-12 media under 37°C. Error bars represent standard deviations (n = 3).

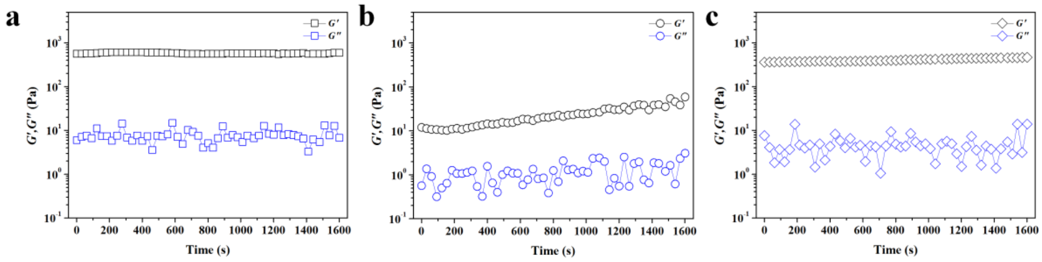

Figure S5 The rheological measurement of self-healing CEC-l-OSA hydrogel. (a) The *G′* of the CEC-l-OSA hydrogel with gelling time of 24 h (□). (b) The *G′* of the CEC-l-OSA hydrogel with gelling time of 30 min (◊). (c) The *G′* of the CEC-l-OSA hydrogel with healing time of 5 min after injection (○).

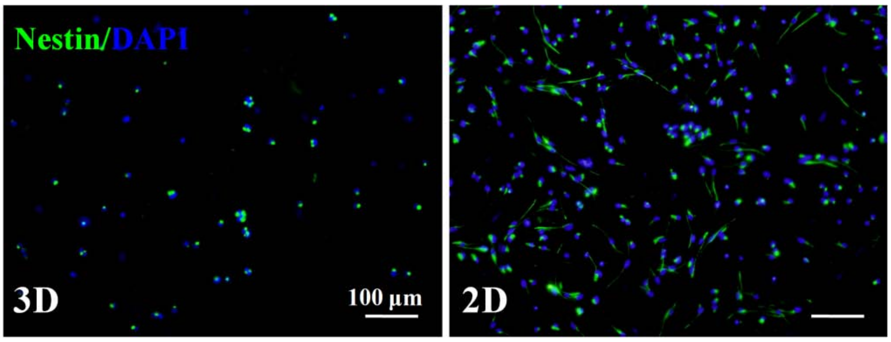

Figure S6 The stemness identification of 3D and 2D cultured NSCs. The expression of nestin marker of the 3D encapsulated NSCs cultured in CEC-l-OSA hydrogels (C_c= 0.02 g/mL), and the NSCs 2D cultured on tissue culture plastics. All the cell nucleus dyed by DAPI are blue color, and the cells immunostained with nestin marker are in green color.

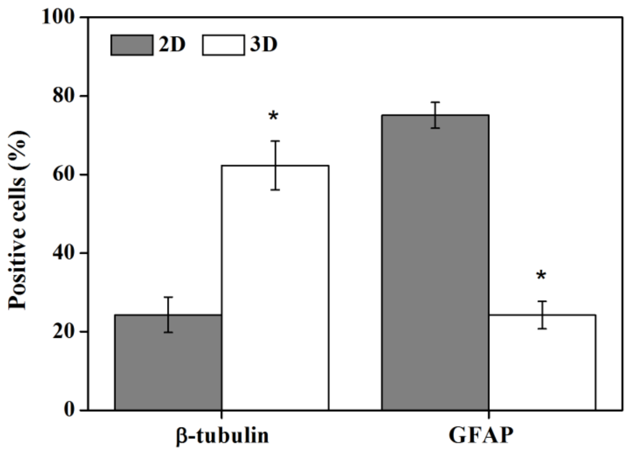

Figure S7 The positive cells expression of β-tubulin and gfap marker of NSCs cultured on 2D TCPS and encapsulated in CEC-l-OSA hydrogels (C_c = 0.02 g/mL) for 9 days. * symbol indicated the significant differences ($p < 0.05$) between 2D and 3D cultures. Error bars represent standard deviations (n = 3).

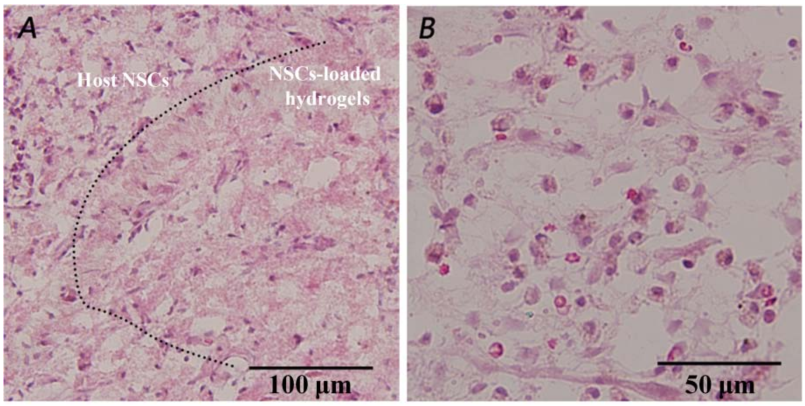

Figure S8 (A) Histological sections of NSCs-loaded hydrogels injected into the mouse focal cerebral ischemia after a week stained with H&E (Hematoxylin-Eosin, the left side of the curve was the host cells and the right side was the NSCs-loaded hydrogels, Scale bar: 100 μm). (B) The high-magnification images of the right side NSCs-loaded hydrogels (Scale bar: 50 μm).
